# Development of an artificial intelligence model to identify duodenal polyps in patients with familial adenomatous polyposis

**DOI:** 10.1016/j.igie.2025.09.009

**Published:** 2025-09-23

**Authors:** Daniel Schupack, Shubham Sood, Jeffrey Fetzer, Shradha Shalini, Shivaram P. Arunachalam, John B. League, Cadman Leggett, Lisa Boardman, Nayantara Coelho-Prabhu

**Affiliations:** Mayo Clinic Division of Gastroenterology and Hepatology, Rochester, Minnesota, USA

## Abstract

**Background and Aims:**

Precancerous duodenal polyps can be subtle in familial adenomatous polyposis (FAP). Artificial intelligence models can detect gastrointestinal (GI) tract pathology; thus, we aimed to develop a model to identify duodenal polyps in FAP patients.

**Methods:**

Images of duodenal polyps from upper GI endoscopic surveillance in FAP patients were obtained. Polyps were manually annotated (Label Studio; HumanSignal, San Francisco, Calif, USA), and the nnU-Net framework (Applied Computer Vision Lab of Helmholtz Imaging and the Division of Medical Image Computing at the German Cancer Research Center, Heidelberg, Germany) was used to automate polyp segmentation. Images were randomly divided into training, validation, and test sets (80%, 10%, and 10%, respectively). Primary performance metric was Dice coefficient (score 0-1). Manual counting was also used to compare the model's ability to identify polyps.

**Results:**

Mean polyps per image by manual count was 4.2 (standard deviation [SD] = 4.8) and by prediction model 4.0 (SD = 4.7). Sixty of 87 images (69.0%) had 0 missed polyps, 74 of 87 (85.1%) missed a maximum of 1 polyp, and 83 of 87 (95.4%) missed a maximum of 4. Mean missed polyps per image was 0.9 (SD = 2.3), and the model identified 287 of 365 (78.6%) polyps. Forty of 87 (46.0%) had falsely identified polyps (mean 0.8; SD = 1.0). Missed polyps were smaller than identified polyps (mean 73.3 [SD = 45.3] versus 233.5 [SD = 274.5] pixel diameter, respectively). There was a complete match (no missed or false positive) in 33 of 87 images (37.9%). Dice coefficient was 0.73.

**Conclusions:**

A model to identify duodenal polyps in FAP was successfully created. Although the Dice coefficient is modest compared with that of colon polyp models, duodenal anatomy creates a challenging background for human and computer detection. Rate of polyp detection, likely a superior marker of goal achievement, was >75%, with a low false polyp rate (mean <1/image). This prototype model is the first step toward a refined algorithm to assist in identification of duodenal polyps with a need for larger prospective studies.

## Introduction

Individuals with familial adenomatous polyposis (FAP) are prone to gastrointestinal (GI) tract polyp formation, which is most severe in the colon but also affects the stomach and proximal small bowel.^1^, ^2^, ^3^ Although penetrance is nearly 100%,^1^ there is variability in the degree of polyposis in individuals with this condition, likely because there are numerous adenomatous polyposis coli (APC) gene variants involved.^4^ For most, colon cancer risk is severe enough that prophylactic colectomy is the standard of care.^5^^,^^6^ With this leading to better management of colonic disease and lowered colon cancer risk, it is increasingly important to improve cancer prevention and detection in other parts of the GI tract.

The duodenum is the next-most common site of cancer in FAP.^5^^,^^6^ Spigelman classification, using duodenal polyp features (number, size, histology, dysplasia), helps guide follow-up,^7^ but true benefit from use of this tool in cancer prevention and survival is unclear, as duodenal polyp detection is difficult, and appropriate management of polyps when they are found is not standardized.

Artificial intelligence (AI) models are being studied and used within endoscopic practice, with models created for detection of colon polyps^8^, ^9^, ^10^ as well as pathology in the upper GI tract, including villous atrophy, inflammation, and angioectasias.^11^, ^12^, ^13^, ^14^, ^15^, ^16^, ^17^, ^18^, ^19^ Models have also been successful in identifying protruding small-bowel lesions on video capsule endoscopy,^20^^,^^21^ leading to our hypothesis that AI may be able to assist in duodenal polyp detection and clinical management in FAP. Given the differing anatomy and mucosal properties between the colon and small bowel, known models to detect colon polyps were not expected to be successful or appropriate for use in this scenario; thus, we pursued development of a novel polyp-detecting model specific to the proximal small bowel.

## Methods

**Figure 1:**
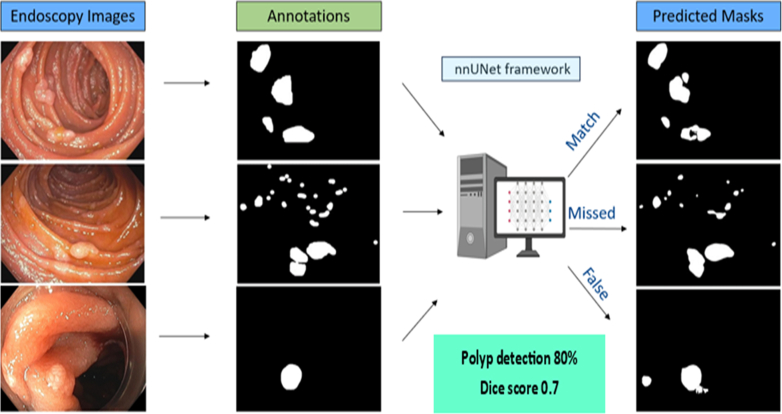
Study flow diagram showing examples of included duodenal polyp images, areas defined as a polyp by study team review (annotations), and areas defined as a polyp by the algorithm on test images after it was trained (predicted masks) using the nnU-Net framework. In this study, 80% of images were used for training, 10% for validation, and finally 10% for the test set. Polyp detection rate by the model was roughly 80% for the test set of images, with a Dice score of 0.7.

This study was approved by Mayo Clinic Institutional Review Board (approved November 24, 2021). FAP patients receiving care at our institution from 1989 to 2023 were identified using diagnosis codes for “familial adenomatous polyposis” and “polyposis,” by searching for patients within our institution who were prescribed the medication Sulindac, and through review of the GI Neoplasia Clinic schedule (2021-2023) to identify as complete a cohort of individuals with FAP as possible. Demographic and clinical data were abstracted from the electronic medical record. Archived images of the upper GI tract mucosa from endoscopic procedures that these patients underwent for clinical purposes were retrospectively obtained and reviewed by a single author (D.S.), retaining only small-bowel images and removing low-quality (blurred), artifact containing (text, tools, blood, cautery markings, dye), and narrow-band imaging pictures. Images from endoscopic ultrasonography (EUS) and endoscopic retrograde cholangiopancreatography (ERCP) procedures were included if they did not obtain features of low-quality images as noted. The immediate goal of this study was to create a duodenal polyp–detecting AI algorithm; thus, to avoid bias and confusion, images containing the ampulla were removed, with the understanding that future studies and refinement of the created algorithm would be required to get to a clinically relevant and effective tool. Images were annotated to mark polyps using Label Studio (HumanSignal, San Francisco, Calif, USA)^22^ by a single author (D.S.), a gastroenterologist with expertise in FAP through advanced GI neoplasia fellowship training and ongoing clinical and research work within this realm. The nnU-Net (isensee2021nnu) (Applied Computer Vision Lab of Helmholtz Imaging and the Division of Medical Image Computing at the German Cancer Research Center, Heidelberg, Germany),^23^ a self-configuring method for deep learning–based image segmentation, was used to automate segmentation of polyps. The data set was randomly divided into training, validation, and test sets (80%, 10%, to 10% split). The primary metric of model performance was Dice coefficient, from 0 (no agreement) to 1 (perfect agreement), gauging similarity between model predictions (what was defined as polyp by the AI algorithm after it was trained) and ground truth annotations (what was defined as polyp by the research team) (Fig. 1). Manual counting of polyps was also used to evaluate the model, completed by 1 author (D.S.).

### Model training and configuration

To automate the segmentation of duodenal polyps from endoscopic images, we developed and evaluated 2 convolutional neural network–based models: a standard U-Net (University of Freiburg, Freiburg, Germany) and the self-configuring nnU-Net framework.^23^ Both models were trained and tested on a data set comprising high-resolution duodenal endoscopy images with expert-annotated masks.

All images were resized to a uniform resolution of 224 × 224 pixels. To enhance model robustness against variations in polyp size, shape, and orientation, we applied data augmentation techniques including horizontal and vertical flipping. Additionally, we implemented a series of image-filtering methods to enhance polyp visibility and edge clarity. Median filtering with multiple kernel sizes was used to reduce random noise while preserving structural features, and the Perona-Malik anisotropic diffusion filter was applied to suppress background textures and sharpen polyp boundaries.

The U-Net model followed a typical encoder-decoder architecture. It was implemented using TensorFlow (Google, Mountain View, Calif, USA) framework and trained using a composite loss function combining binary cross-entropy and optimized via the Adam (Kingma & Ba, University of Toronto, Toronto, Canada) optimizer. Hyperparameters, including learning rate, batch size, and number of epochs, were tuned using a validation subset of the training data, with early stopping used to prevent overfitting.

In parallel, we used the nnU-Net framework, which is designed to automatically adapt its architecture, preprocessing pipeline, and training strategy based on data set characteristics. Without requiring manual configuration, nnU-Net determined the optimal network depth, input patch size, learning rate schedule, and postprocessing methods tailored to our data set.

Both models were trained and evaluated on the same data set. Performance was assessed using the Dice similarity coefficient. During training, we optimized a soft (pseudo) Dice objective that compares the model's per-pixel probabilities directly with the binary ground truth, avoiding thresholding at uncertain boundaries. For evaluation on the held-out test set, we report the classical (hard) Dice computed on binary predictions obtained by applying a single, prespecified threshold to the probability map; this threshold was fixed on the validation set. Because soft Dice preserves information about near-threshold probabilities, whereas hard Dice discards them at binarization, soft Dice values can be higher than the corresponding hard Dice values for the same prediction, especially at fuzzy edges. When model outputs are strictly binary, the 2 measures coincide.

### Comparative analysis

Results were compared against vanilla U-Net to benchmark performance against state-of-the-art methods, providing context of model capabilities and highlighting areas for future research/development. A “vanilla” U-Net refers to the foundational deep learning architecture specifically designed for image segmentation. In contrast, nnU-Net is an automated framework that leverages this U-Net architecture but intelligently self-configures and optimizes all the parameters based on the characteristics of the input data. Regarding the median filter kernel configurations, a median filter is a digital image-processing technique used to reduce noise by replacing each pixel's value with the median of its neighbors within a defined window. Here, a “kernel” refers to a small, predefined matrix or window of numerical values. This kernel conceptually slides across every pixel of an image. At each position, the values of the pixels within the image region covered by the kernel (its “neighborhood”) are used to perform a specific calculation. For a median filter, the kernel defines the exact size and shape of the local area from which the median pixel intensity value is computed. This computed median value then replaces the original pixel's intensity value at the center of the kernel, effectively reducing noise. Therefore, the kernel size directly refers to the dimensions of this small window (eg, 3 × 3 pixels, 5 × 5 pixels), with larger kernels encompassing a wider neighborhood for the filtering operation.

Vanilla U-Net and nnU-Net Dice scores for the median filter kernel are presented in Table 1.

**Table 1:** Comparison of neural network architectures for medical image segmentation (U-Net and nnU-Net models)

Filter size	Dice score	
	Vanilla U-Net	nnU-Net
No filter	0.452	0.730
3 × 3 filter (kernels)	0.441	0.712
5 × 5 filter (kernels)	0.403	0.705
7 × 7 filter (kernels)	0.394	0.695
9 × 9 filter (kernels)	0.400	0.687
11 × 11 filter (kernels)	0.368	0.664
13 × 13 filter (kernels)	0.378	0.632

## Results

**Figure 2:**
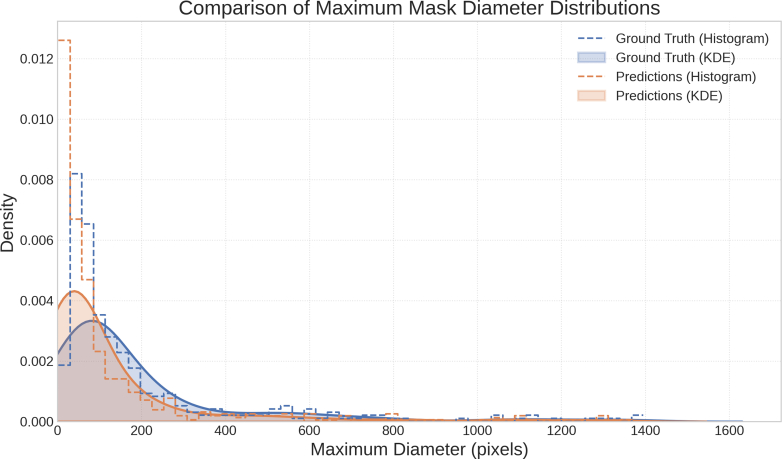
Size distribution in pixel diameter of ground truth annotations (*blue*) and model-predicted masks (*orange*) of duodenal polyps in the study images. The *y* axis represents probability density. For the histogram, the height of each bar is scaled such that the area of the bar corresponds to the proportion of observations falling within that bin. For the kernel density estimate (KDE), the curve represents the estimated probability density function, where the area under the curve over an interval gives the estimated probability of an observation falling within that interval. In both cases, the total area under the normalized histogram and the KDE curve is equal to 1, signifying the full distribution of the data.

Of 498 FAP patients in our database, 371 had at least 1 upper GI tract endoscopic evaluation (esophagogastroduodenoscopy, ERCP, EUS) completed at our institution. A total of 2547 procedures were reviewed, with 870 high-quality duodenal polyp images obtained from these. Images were randomly divided for training (80%), validation (10%), and model testing (10%), with the 87 test set images containing 365 polyps (mean polyp diameter 196.3 pixels; standard deviation [SD] = 250.7) (Fig. 2).

**Figure 3:**
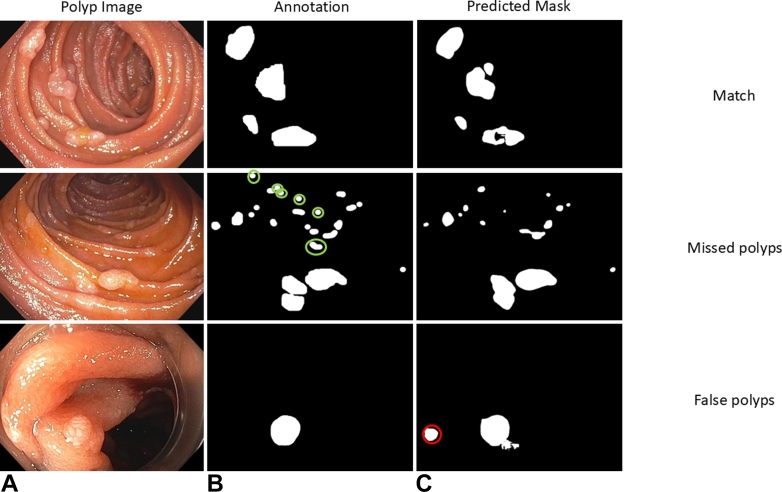
Representative duodenal polyp images (**A**) annotations (ground truth; **B**), and predicted masks (**C**) showing well-matched prediction, missed polyps, and falsely identified polyps. Missed polyps are *circled in green* and false polyps *circled in red*.

**Figure 4:**
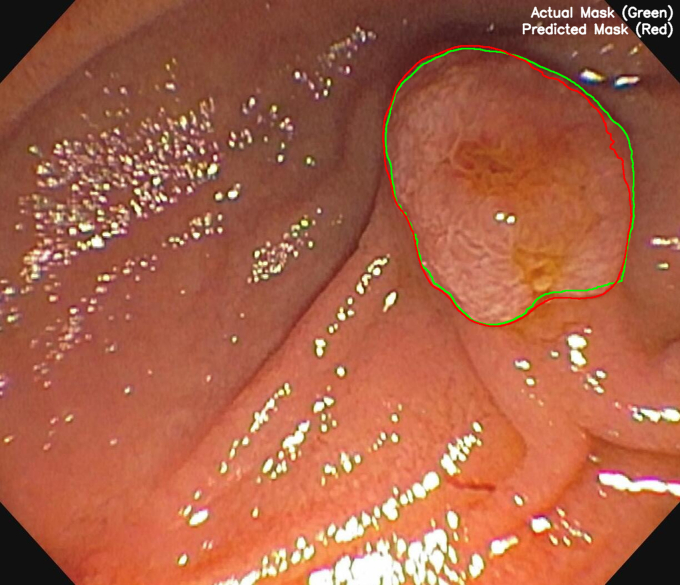
Ground truth (actual mask) noted by the *green outline* and algorithm prediction (predicted mask) noted by the *red outline* superimposed on duodenal polyp original image.

**Figure 5:**
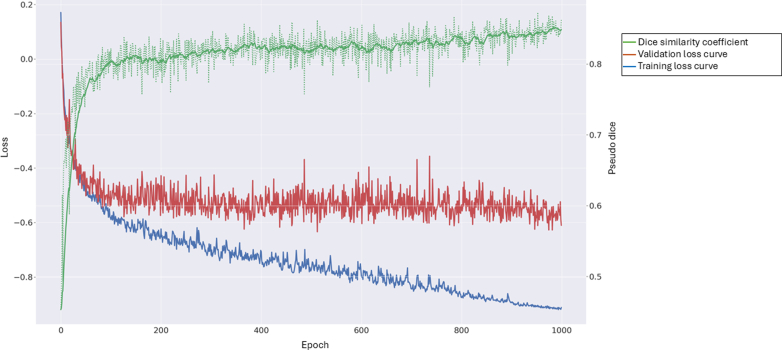
Training loss, validation loss and Dice curves for the polyp-detecting model. This figure illustrates the model's learning progress over successive training epochs. An epoch represents 1 complete pass through the entire training data set, during which the model's parameters are updated. The Training Loss (*blue line*) measures the model's error on the data it is actively learning from, whereas the Validation Loss (*orange line*) tracks the model's error on an independent, unseen validation data set. A sharp decrease in both loss curves, particularly the validation loss, early in training indicates rapid improvement in the model's ability to learn from the data and generalize to unseen examples. The subsequent plateau of the validation loss signifies that the model has largely converged and reached a stable level of performance on new data. At this point, additional epochs do not substantially improve its generalization ability, suggesting a suitable point for early stopping to prevent overfitting and optimizing computational resources. Although the standard Dice similarity coefficient measures the overlap between 2 distinct binary shapes (like a perfectly outlined polyp by a human vs artificial intelligence [AI]), AI models for segmentation typically output a probability map—assigning a continuous confidence level (eg, 0%-100%) to each pixel, indicating its likelihood of belonging to a structure. Pseudo Dice (or soft Dice) directly compares this continuous probability map from the AI with the ground truth outline, rather than first forcing the AI's output into a rigid binary shape by picking an arbitrary cutoff threshold. This approach offers a more nuanced evaluation of the AI's performance, as it accounts for the model's varying confidence across a region, particularly at fuzzy boundaries, thereby providing a truer reflection of overall agreement between the AI's probabilistic prediction and the actual region of interest.

**Figure 6:**
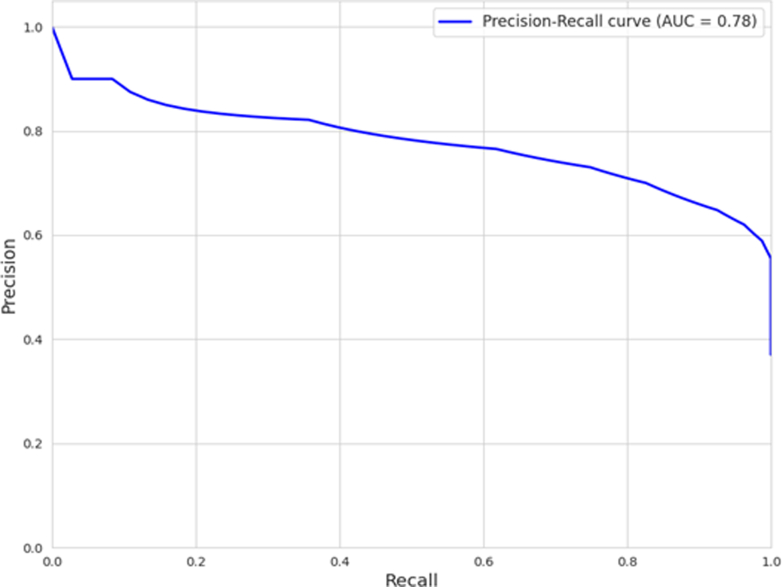
Precision-recall curve for Dice score of the prediction model. In the context of a precision-recall curve, precision quantifies the proportion of correctly identified positive predictions among all the positive predictions made by the model, whereas recall measures the proportion of correctly identified positive predictions among all actual positive instances. The area under the curve (AUC) for a precision-recall curve provides a single scalar value representing the aggregate performance of the model across all possible classification thresholds, indicating its overall ability to achieve high precision and high recall simultaneously.

Per manual count, there were 4.2 polyps per image (SD = 4.8), and by prediction model, 4.0 polyps per image (SD = 4.7). On 60 of 87 images (69.0%), the model missed 0 polyps, on 74 of 87 (85.1%) images, a maximum of 1 polyp was missed, and on 83 of 87 (95.4%) images, a maximum of 4 polyps were missed, with a mean of 0.9 missed polyps per image (SD = 2.3) and 287 of 365 (78.6%) polyps identified overall (Figs. 3 and 4). The mean diameter of the polyps accurately identified was 233.5 pixels (SD = 274.5) versus 73.3 pixels (SD = 45.3) for missed polyps (*P* < .001). In the 4 test images with more than 4 missed polyps, the mean polyp number was 19.8 (SD = 6.4). Falsely identified polyps occurred in 40 of 87 (46.0%) images, with a mean of 0.8 polyps per image (SD = 1.0). There was a complete match (no missed and no false-positive polyps) in 33 of 87 images (37.9%). Dice coefficient was 0.73 (95% confidence interval, 0.673-0.775, *P* < .001) for the prediction model on the test images (Figs. 5 and 6).

Polyp detection differed in images with low polyp burden (≤5 polyps) versus high burden (>5 polyps), with 126 of 142 (88.7%) polyps identified in low burden versus 161 of 223 (72.2%) polyps in high burden (*P* < .001), and 65 of 67 (97.0%) images with a maximum of 1 missed polyp in low burden versus 9 of 20 (45.0%) in high burden. The model had a higher rate of false polyps in high burden (1.3/image [SD = 1.0]) versus low burden (0.6/image [SD = 1.0]) (*P* = .01).

## Discussion

We present a novel image-based deep learning model to identify duodenal polyps in patients with FAP. The model detected 79% of polyps, with 85% of images having 0 polyps or 1 polyp missed. Most missed polyps were small on the image (mean pixel diameter >3 times larger in identified polyps vs missed), suggesting that the model excelled with more substantial and likely more clinically relevant polyps. We recognize the limitation that the size of polyps in the data set is not always accurate because still images were used, making polyps farther from the lens appear smaller. Therefore, polyp size was measured by image pixel number in this study and not millimeters, the usual clinical standard, because this was felt to more accurately compare polyp size on the retrospectively obtained still images with no standard distance of images from the polyps themselves. Because the use of the standard millimeter measurement would inaccurately depict the true size of polyps, we feel it is relevant to describe results in this manner using pixels. As we hope to expand and improve the algorithm with future endoscopic video input, allowing for more accurate polyp size identification, we anticipate size estimation to significantly improve and to move into a more clinically relevant description of polyp size with our future work.

Dice score (0.73) is modest in relation to models for colon polyps but likely related to the variable background of small-bowel mucosa, rendering adenoma detection more difficult, and we feel that the absolute polyp detection rate of >75% represents a successful detection model. Beyond this, we also attempted model development with a recent pretrained model (ASPS [Augmented Segment Anything Model for Polyp Segmentation]),^24^ with a substantially lower Dice score (0.42); thus, we feel that model creation was rigorous, and we identified the most effective model within current segmentation methods.

Despite successful model creation, there remain issues to address before practical clinical use of this AI algorithm. We acknowledge lack of ampullary images as a limitation, especially given difficulty in clarifying polyp presence in this area. With ERCP, models have been somewhat successful in identifying the ampulla to assist in difficult cannulations.^25^ As one of the next steps, we plan to develop an algorithm to identify the ampulla itself, and secondarily adenomatous tissue there, so that this can be incorporated with our current polyp-detecting model to work toward a successful clinical tool. Our model also did not perform as well with large polyp burden. Some of this issue lies within the use of still images in our model, because images with high polyp burden often contained polyps in the background of the images. With increased data abstraction of images through procedural video recordings, we aim to improve the algorithm to be more effective in this scenario. Although lower success with high polyp burden is not ideal, this may be of less concern regarding the ability of AI to guide FAP clinical care, as the biggest clinical assistance long term would likely relate more to polyp risk stratification than to detection itself.

The ultimate long-term goal we are seeking is to harness the power of AI to help with management of upper GI tract pathology in patients with FAP in real-time clinical care scenarios so that more effective and efficient care can be delivered. Polyp detection is the first step toward this goal, and our algorithm was able to do this modestly well. To refine our algorithm in detecting polyps and differentiating among normal, abnormal, and artifactual lesions, the next steps will involve including visual data from video recordings with polyp annotation by multiple expert endoscopists and developing an algorithm to detect the ampulla. To truly improve upper GI tract FAP management endoscopically, working toward real-time identification of high-risk polyps for selective polypectomy is our goal and represents an avenue for future projects. With the overall low rate of high-grade dysplasia and malignancy in individual polyps even in FAP, long-term prospective studies are needed to work toward developing a polyp risk–stratifying AI algorithm. Prospective studies using procedural video recording will allow for matching histology to polyp visualization, which is not feasible retrospectively and will be necessary to achieve the goal of improving endoscopic management of duodenal pathology in FAP with AI.

## Patient consent

This article does not discuss individual patients, so no consent was needed.

## Disclosure

All authors disclosed no financial relationships.
